# Inhibition of *Arabidopsis* chloroplast β-amylase BAM3 by maltotriose suggests a mechanism for the control of transitory leaf starch mobilisation

**DOI:** 10.1371/journal.pone.0172504

**Published:** 2017-02-22

**Authors:** Jing Li, Wenxu Zhou, Perigio Francisco, Russell Wong, Dongke Zhang, Steven M. Smith

**Affiliations:** 1 School of Plant Biology, The University of Western Australia, Western Australia, Australia; 2 Centre for Energy, Faculty of Engineering, Computing and Mathematics, The University of Western Australia, Western Australia, Australia; 3 School of Biological Sciences, University of Tasmania, Tasmania, Australia; Universidade Federal de Vicosa, BRAZIL

## Abstract

Starch breakdown in leaves at night is tightly matched to the duration of the dark period, but the mechanism by which this regulation is achieved is unknown. In *Arabidopsis* chloroplasts, β-amylase BAM3 hydrolyses transitory starch, producing maltose and residual maltotriose. The aim of the current research was to investigate the regulatory and kinetic properties of BAM3. The BAM3 protein was expressed in *Escherichia coli* and first assayed using a model substrate. Enzyme activity was stimulated by treatment with dithiothreitol and was increased 40% by 2–10 μM Ca^2+^ but did not require Mg^2+^. In order to investigate substrate specificity and possible regulatory effects of glucans, we developed a GC-MS method to assay reaction products. BAM3 readily hydrolysed maltohexaose with a K_m_ of 1.7 mM and K_cat_ of 4300 s^-1^ but activity was 3.4-fold lower with maltopentaose and was negligible with maltotetraose. With maltohexaose or amylopectin as substrates and using [UL-^13^C_12_]maltose in an isotopic dilution method, we discovered that BAM3 activity is inhibited by maltotriose at physiological (mM) concentrations, but not by maltose. In contrast, the extracellular β-amylase of barley is only weakly inhibited by maltotriose. Our results may explain the impaired starch breakdown in maltotriose-accumulating mutants such as *dpe1* which lacks the chloroplast disproportionating enzyme (DPE1) metabolising maltotriose to glucose. We hypothesise that the rate of starch breakdown in leaves can be regulated by inhibition of BAM3 by maltotriose, the concentration of which is determined by DPE, which is in turn influenced by the stromal concentration of glucose. Since the plastid glucose transporter pGlcT catalyses facilitated diffusion between stroma and cytosol, changes in consumption of glucose in the cytosol are expected to lead to concomitant changes in plastid glucose and maltotriose, and hence compensatory changes in BAM3 activity.

## Introduction

The synthesis and breakdown of transitory starch in leaves is very tightly regulated throughout the diurnal cycle. In *Arabidopsis thaliana* it accumulates linearly during the day then declines linearly during the night such that all the starch accumulated during the day is consumed during the night [1]. Plants grown under short photoperiods synthesise starch more rapidly in the light than do plants grown in long photoperiods, and then break it down more slowly during the long night, so that carbon is available for growth throughout the night in both cases [2]. When plants are grown in long days then subjected to a short day by premature transfer to darkness, they can respond immediately by establishing a slow rate of dark breakdown so that the available starch will be broken down at a constant rate and will last for the period of the extended night [1–4]. The mechanisms by which such tight control of rates of starch breakdown is achieved are unknown [5, 6].

Progress in elucidating the pathway of starch breakdown in Arabidopsis leaves has identified the key enzymes required for the process during normal diurnal growth. Phosphorylation and subsequent dephosphorylation of amylopectin at the surface of the starch granule is required, catalysed by glucan water dikinase (GWD), phosphoglucan water dikinase (PWD) and a dual-specificity phosphoglucan phosphatase (DSP, also known as SEX4 and LSF2) [7–13]. It is hypothesised that phosphorylation and dephosphorylation serve to disrupt the crystalline nature of the starch granule and so allow access by glucan hydrolysing enzymes [5, 11, 12]. Hydrolysis of α-1,4-glucan linkages by β-amylase BAM3 removes maltose residues from the non-reducing ends of accessible glucan chains, and isoamylase ISA3 hydrolyses α-1,6-linkages to debranch the amylopectin [14, 15]. Both BAM3 and ISA3 are active at the starch granule surface [11, 12, 15, 16], but BAM3 can potentially also act upon soluble glucans released by ISA3 [1, 11]. While maltose is the primary product of starch breakdown, maltotriose is also produced as a residual product from glucan chains with odd-numbers of glucose residues [14]. Maltose is exported from the plastid by the maltose exporter (MEX1) while maltotriose is converted to maltopentaose and glucose by plastidial disproportionating enzyme (DPE1) [17, 18]. The maltopentaose can be further broken down by BAM3 to maltose and maltotriose. Thus the concerted action of DPE1 and BAM3 converts maltotriose to maltose and glucose for export by MEX1 and plastidial glucose transporter (pGlcT), respectively [14, 17–20]. BAM3 therefore occupies a central position in the breakdown of starch in leaves at night and is a potential point of control.

The conditions in the chloroplast stroma differ significantly between light and dark and can potentially regulate enzyme activity. In response to light the stroma experiences a transient increase in Ca^2+^ concentration as well as sustained increases in Mg^2+^ concentration and pH [21–23]. We have previously determined that BAM3 has a broad pH optimum with activity peaking at pH 6.0, declining at higher values up to pH 8.0 [16], and this is supported by more recent studies [24]. This observation suggests that BAM3 is more active in the dark than in the light since the pH of the chloroplast stroma during the night is around 7 increasing to 8 in the day [4]. There is also an increase in availability of reducing equivalents from the photosynthetic electron transport chain and many enzymes have been shown to be activated by reduction including ADPglucose pyrophosphorylase (AGPase), GWD1 and plastidial α-amylase AMY3 [6, 25, 26, 27]. Redox regulation of BAM3 has not been reported, but BAM1, another plastidial β-amylase, is subject to activation by reduction [28]. However BAM1 is not essential for transitory starch breakdown in mesophyll tissue under normal conditions. It is believed that BAM1 plays an important role in starch breakdown in guard cells in the light [29]. Another potential means of control of amylolytic enzymes is by product inhibition. For example, it has been reported that maltose is a weak competitive inhibitor (K_i_ 20–30 mM) of barley seed β-amylase while glucose at 69 mM and maltotriose at 28 mM show no inhibitory effects [30]. However, possible regulation of BAM3 by metal ions, redox status or product inhibition has not been reported. Here we show that BAM3 displays relatively little response to Ca^2+^ and Mg^2+^ concentrations but activity requires dithiothreitol. Surprisingly, it is inhibited by maltotriose but not maltose, suggesting a novel means for the control of starch breakdown in leaves.

## Materials and methods

### Materials, reagents and instrumentation

Enzyme substrates and inhibitors with highest available purity were purchased from Sigma unless otherwise indicated. Carbohydrates used were glucose (G827), maltose (Fluka 63419), maltotriose (M8378), maltotetraose (M8253), maltopentaose (M8128) maltohexaose (M9153), [UL-^13^C_12_]maltose (Omicron Biochemicals, MAL-002), potato amylopectin (Fluka, 10118), sucrose (S0389), D-(+)-cellobiose (C7252), D-(+)-trehalose (T9531) and isomaltose (I7253), D-(+)-melezitose (63620) and Raffinose (83400). GC-MS (6890 GC coupled with 5975N MSD) was from Agilent, equipped with Varian Factor 4 VF-5ms capillary column.

### Enzyme preparation

Expression of recombinant BAM1 and BAM3 proteins in *Escherichia coli* and purification were described previously [16], barley BAM was obtained from Sigma (A7130), and the lyophilized protein powder was reconstituted according to the manufacture’s manual. All enzymes were kept at—80°C, and thawed on ice before experiments.

### Effects of divalent cations and dithiothreitol on BAM3 activity

Activities of β-amylases were assayed using Βetamyl kits (Megazyme, Bray, Ireland) according to manufacturer’s instructions [14]. Enzymes were incubated with the artificial substrate *p*-nitrophenyl maltopentaoside (PNPG5) at 2.5 mM in 0.1 M maleic acid assay buffer, pH 6.2, at 40°C for 10 min, then colorimetric readings were taken. For the study of regulation by divalent cations BAM3 activity was measured at concentrations of Ca^2+^ from 1 μM to 40 mM (using CaCl_2_) and Mg^2+^ from 1 mM to 50 mM (using MgCl_2_). Redox modulation of enzyme activities was also assayed using the Βetamyl kit, but BAM3, BAM1 and barley BAM were first pre-treated with 20 mM DTT either in reduced form (Sigma: D9779) or oxidized form (Sigma: D3511) at 37°C for 1 h [28], before adding to the assay. Student’s *t*-test was performed by QuickCalcs, GraphPad Software online (http://www.graphpad.com/quickcalcs/ttest1.cfm) for statistical significance of data.

### Gas Chromatography-Mass Spectrometry (GC-MS) method for assaying β-amylase activities

Since the Betamyl assay system is restricted to artificial substrates such as PNPG5, a GC-MS assay method was established to determine amounts of maltose produced from different α-glucan substrates. The different enzyme substrates tested were potato amylopectin (AP), maltotetraose (G4), maltopentaose (G5) and maltohexaose (G6). Assay mixture (50 μL) comprising 50 mM sodium acetate, 5 mM DTT, 5 mM EDTA, pH 6.0, and containing each substrate at a range of concentrations, was pre-incubated at 25°C for 10 min, then 0.0135 μM enzyme was added to the assay mixture with or without potential effectors, and incubated at 25°C for 20 min. The reactions were terminated at 65°C by addition of 500 μL methanol, and sucrose was added as an internal standard prior to GC-MS analysis. The mixture (100 μL) was brought to dryness under vacuum and sugars in the residue were converted to their *trimethylsilyl esters* using *N*,*O*-bis(trimethylsilyl)trifluoroacetamide (BSTFA) with pyridine at 80°C for 30 min. Sugar derivatives were subjected to GC-MS analysis and maltose was quantified based on its total ion current (TIC) peak compared to that of internal standard. GC-MS conditions are as follow: the oven was programmed with an initial temperature of 200°C and held for 1 min, then the temperature was ramped to 340°C at 10°C min^-1^ and held for 5 min. Helium was the carrier gas, with constant flow of 1 ml min^-1^.

The Michaelis-Menten steady-state kinetic parameters K_m_ and V_max_ were calculated from the results of linear regression of Lineweaver Burk plots according to the equation 1/v = K_m_/(V_max_ [S]) + 1/V_max_. The inhibition type was determined by Lineweaver Burk plots, and the K_i_ of the inhibitor was calculated by non-linear regression using Least Squares Method to fit assay results to the equation: v = V_max_/((K_m_/S) (1+I/K_i_))/[1+I/(a/K_i_). In all cases, the R^2^ value was greater than 0.97. The Lineweaver Burk plots and linear regressions were from Microsoft Excel 2003; the non-linear regression for K_i_ calculation was performed on Sigma plot with enzyme kinetic module.

### Effect of maltose on BAM3 and barley BAM using isotopic dilution GC-MS

The GC-MS method enabled us to distinguish naturally-occurring [^12^C]maltose from [^13^C]labelled maltose and so permitted development of an isotopic dilution method to investigate the effect on enzyme activity of adding [UL-^13^C_12_]maltose (detailed validation of the method has been prepared for a separate manuscript). Briefly, [UL-^13^C_12_]maltose from 0 to 10 mM was added to the assay, then after the reaction, enzymatically produced [^12^C]maltose was quantified using GC-MS as described above. Although the added labelled maltose and enzymatically produced maltose eluted from the GC column at the same time, their mass spectra are different. The enzyme product was quantified based on the signal intensity for fragment of *m/z* = 361, whereas, the [UL-^13^C_12_]maltose gave the same fragment at *m/z* = 367. Quantitation of the *m/z* = 361 fragment in all samples before and after incubation allowed for correction due to minor contamination of [UL-^13^C_12_]maltose and glucan substrates with [^12^C]maltose.

## Results

### Effects of reducing equivalents, Ca^2+^ and Mg^2+^ on BAM3 activity

**Fig 1:**
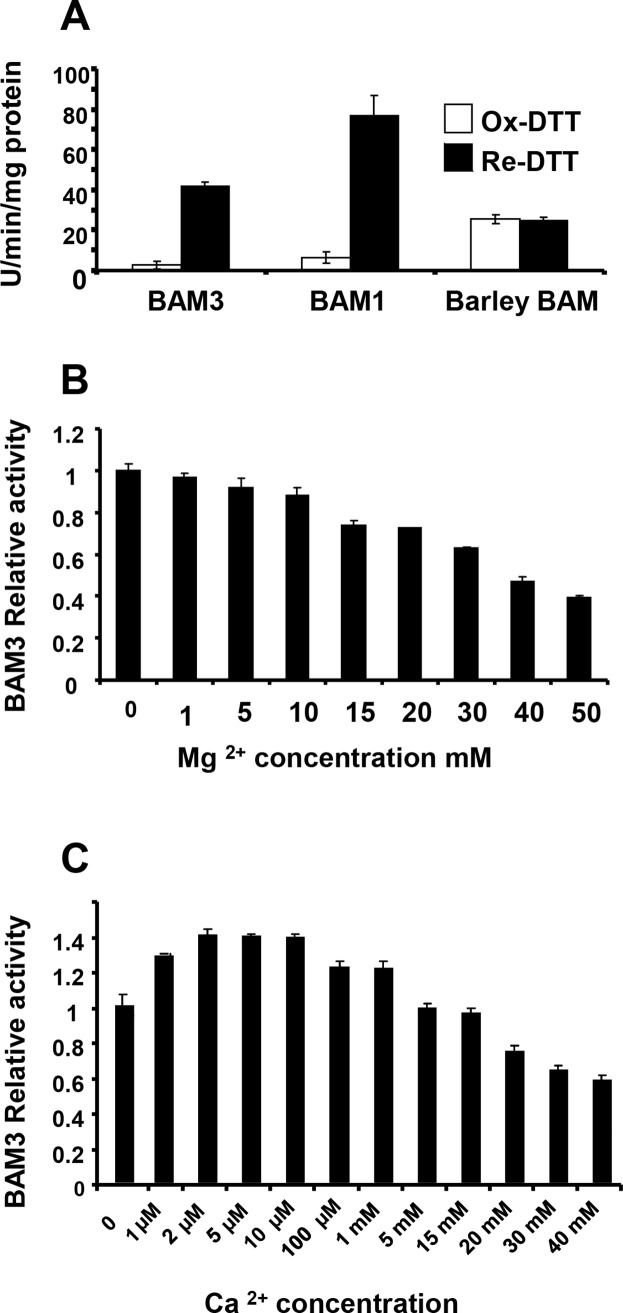
Effects of reducing equivalents, Ca^2+^ and Mg^2+^ on BAM3 activity. **A,** Specific activities of oxidised and reduced forms of BAM1, BAM3 and Barley BAM. Enzymes were incubated at 37°C for 1 h with 20 mM DTT either in oxidised or reduced form before assay. **B:** Activity of BAM3 in the presence of 1 mM to 50 mM Mg^2+^. **C**: Activity of BAM3 in the presence of 1 μM to 40 mM Ca^2+^. All values are the means of three independent assays (+/- SE).

Studies of BAM3 activity employed the protein synthesised in *Escherichia coli* [16]. We initially used the Betamyl assay system with soluble synthetic substrate *p*-nitrophenyl maltopentaoside (PNPG5) and spectrophotometric detection of β-amylase-dependent release of *p*-nitrophenol. BAM3 activity was 17-fold greater when pre-treated with 20 mM dithiothreitol (DTT) in reduced form, relative to pre-treatment with the oxidised form (Fig 1A). For comparison we assayed recombinant BAM1 and commercially-purchased barley seed β-amylase, under the same conditions. We observed that BAM1 activity was also greater (12-fold) following treatment with 20 mM DTT (reduced), as expected from previous reports [28]. In contrast, barley β-amylase activity was not stimulated by pre-treatment with reduced DTT (Fig 1A). Thus BAM3, like BAM1, has the potential to be subject to redox regulation *in vivo* but this possibility requires further investigation. For subsequent experiments, BAM enzymes were prepared and maintained under reducing conditions before being assayed [14, 16]. The activity of BAM3 did not require Mg^2+^ but was markedly inhibited at concentrations above 15 mM (Fig 1B). This is consistent with a K_i_ value of 18.6 mM previously reported for barley [31]. No requirement for Ca^2+^ was observed but BAM3 activity was stimulated by about 40% at concentrations between 2 and 10 μM (Fig 1C). These results suggest that Mg^2+^ and Ca^2+^, together with pH [16], could play a small part in regulating BAM3 activity.

### Substrate specificity of BAM3 and possible effect of maltose on its activity

**Fig 2:**
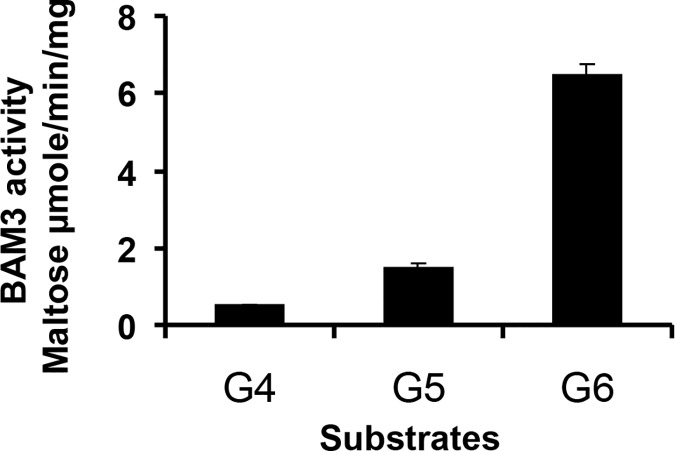
Rates of maltose production by BAM3 with G4, G5 and G6 substrates. The rates of maltose production from 5 mM G4, G5 or G6, were quantified by GC-MS.

To investigate substrate specificity and possible regulatory effects of maltose and other products of starch breakdown on BAM3 activity it was necessary to devise a new assay method since the Betamyl assay system is restricted to artificial substrates such as PNPG5. We developed a method of gas chromatography coupled to mass spectrometry (GC-MS) to assay maltose produced from α-glucan substrates, thus providing flexibility in the choice of substrate. Furthermore the use of MS allowed us to employ [^13^C]maltose as a potential effector molecule and to distinguish it from the [^12^C]maltose product of glucan hydrolysis. Initially we compared maltotetraose (G4), maltopentaose (G5) and maltohexaose (G6) as substrates. It has previously been reported that G4 is a poor substrate for plant β-amylases [32]. We observed that the initial rates of maltose production were very low for G4, three-fold higher for G5 and twelve-fold greater for G6 (Fig 2). Hydrolysis of one G4 molecule by β-amylase generates two of maltose, whereas G5 will liberate only one maltose residue. Thus the rate of reaction of G5 hydrolysis is approximately six-fold higher than for G4. Complete hydrolysis of G6 by β-amylase would liberate three maltose equivalents. However, since hydrolysis of G4 is very slow, even at an initial concentration of 5 mM (Fig 2), we estimate that the amount of maltose generated by hydrolysis of the G4 liberated by the initial action of BAM3 on G6 can be no more than about one twelfth (8%) of the maltose observed which represents 4% of the BAM3 activity. However the BAM3 hydrolysis of G4 would be very much less during the initial reaction. Therefore for subsequent experiments using G5 and G6 substrates we consider maltose production rates to equate to reaction rates.

**Fig 3:**
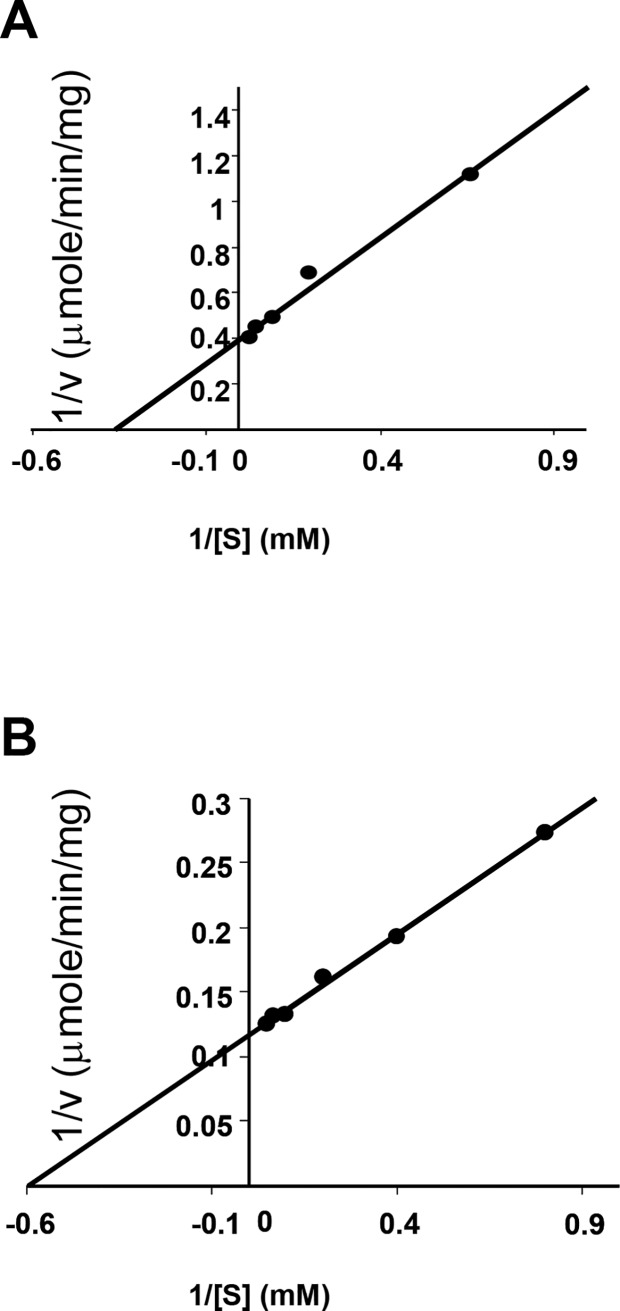
Comparison of BAM3 kinetic parameters using G5 and G6 as substrates. BAM3 kinetic parameters were measured using G5 (**A**) and G6 (**B**) as substrate. Michaelis-Menten and kinetic data were generated using the Sigma-enzyme kinetic program.

To characterise the properties of BAM3, we determined the kinetic parameters of the enzyme using G5 and G6 as substrates (Fig 3A and 3B and Table 1). The data fit Michaelis-Menten kinetics and show that the V_max_ is 3.4 fold greater with G6 than with G5 as substrate. Furthermore, BAM3 has a lower affinity for G5 than for G6 (Table 1). We considered the possibility that residual maltotriose released by the action of BAM3 on G5 could inhibit BAM3 activity and so contribute to the lower activity with G5 than with G6, but assays were conducted to measure initial rates of reaction before any product inhibition would be expected.

**Table 1:** Comparison of BAM3 kinetic parameters using G5 and G6 substrates.

	V_max_	K_m_	K_cat_
	(μmole/min/mg)	(mM)	(1/sec)
G5	2.51	2.77	1258
G6	8.58	1.68	4300

BAM3 was assayed using a range of G5 and G6 substrate concentrations (see Fig 3). The production of maltose was determined by GC-MS and the kinetic parameters were calculated as described in Methods.

**Fig 4:**
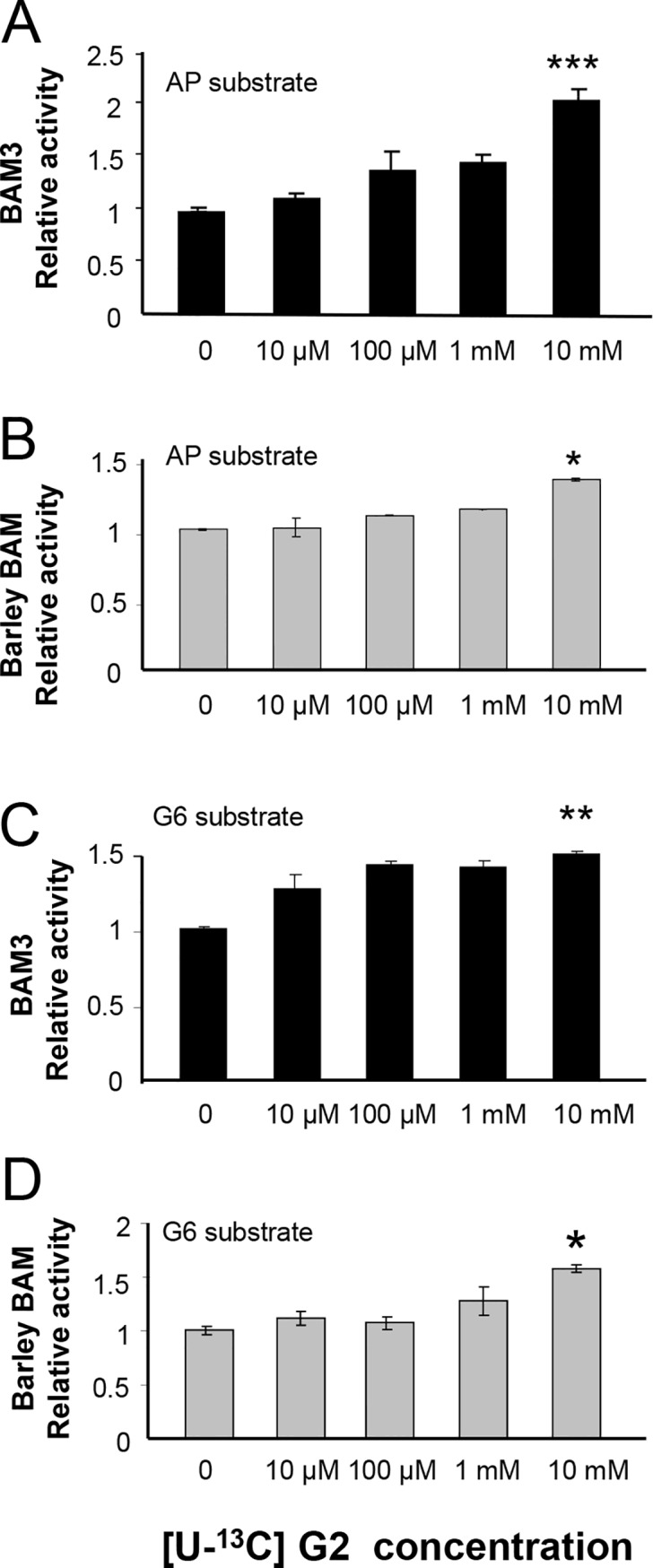
Effect of maltose on BAM3 activity. [UL-^13^C]maltose was added to the assay mix at the concentrations shown and unlabelled maltose as the reaction product was detected by GC-MS. **A:** BAM3 acting on amylopectin (AP) as substrate, **B:** Barley BAM acting on AP, **C:** BAM3 acting on G6, and **D:** Barley BAM acting on G6. Each value is the mean of three independent replicates (+/- SE) and significant differences determined by the Student’s *t*-test are shown (* P > 0.1; ** P > 0.05; *** P > 0.01).

To investigate possible effects of maltose on BAM3 activity we used G6 and potato amylopectin (AP) as alternative substrates, and uniformly-labelled [^13^C]maltose as the effector. Contrary to expectation, BAM3 specific activity increased significantly in response to added maltose (Fig 4A and 4C). Addition of 10 mM maltose stimulated BAM3 activity 1.4-fold with G6 as substrate and 2.2-fold with AP as substrate. Maltose has been reported to be a weak competitive inhibitor of barley β-amylase with a K_i_ of 20 to 30 mM when assayed using the Somogyi-Nelson method [30]. We therefore tested the barley enzyme using our new assay system and observed similar results to those seen with BAM3. Addition of 10 mM maltose stimulated barley β-amylase activity approximately 1.3 fold with AP and 1.5-fold with G6 (Fig 4B and 4D).

To determine if the small stimulatory effect of maltose on BAM3 is specific for this disaccharide we also tested other disaccharides including sucrose, isomaltose (α-1,6-linked glucosyl units rather than α-1,4-linked), trehalose (α-1,1-linked glucosyl units) and cellobiose (β-1,4-linked glucosyl units) but none of these had a significant effect on activity (S1A Fig). To further investigate isomaltose we increased the concentration up to 50 mM and although a small stimulation was suggested, this was not statistically significant (S1B Fig).

We conclude from these studies that maltose does not inhibit either β-amylase, but instead it tends to increase activity, potentially under physiological conditions. We also tested the effect of glucose on BAM3 and barley β-amylase activities and found that up to 50 mM it had no significant effect on either enzyme when using G6 as substrate (S2 Fig).

### Inhibitory effect of maltotriose on BAM3

**Fig 5:**
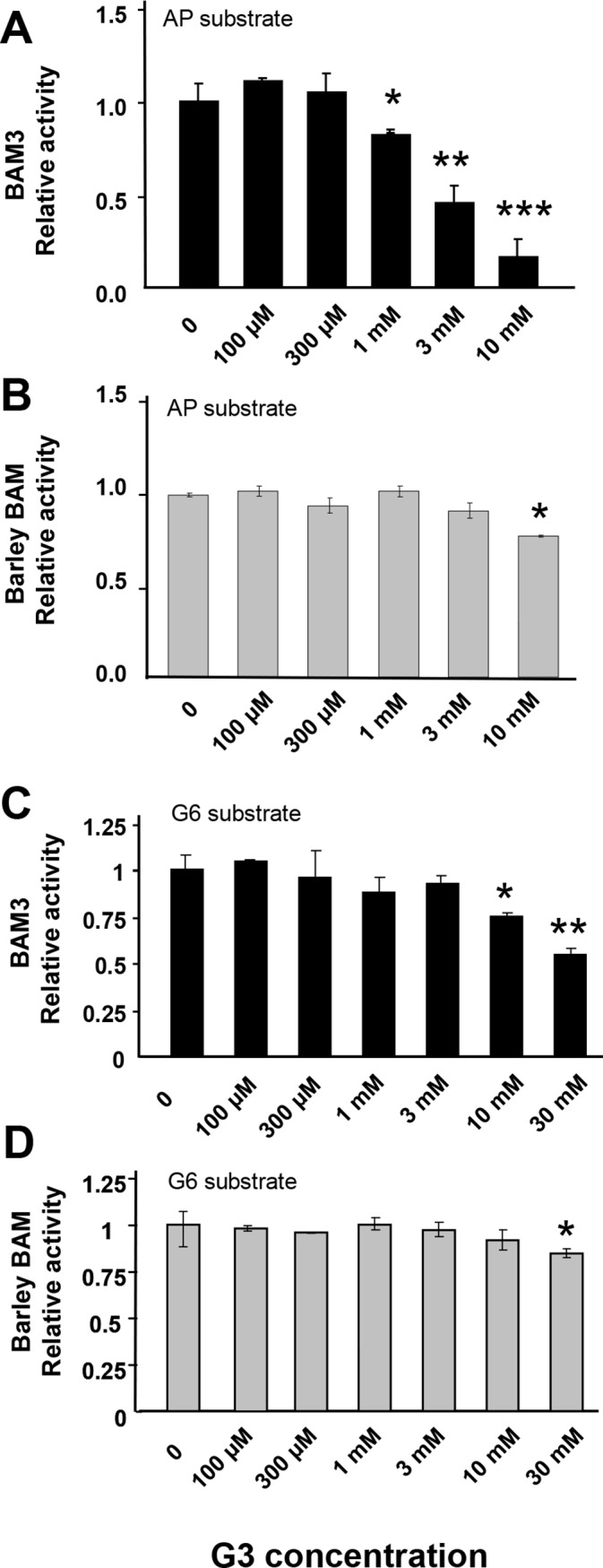
Inhibitory effect of maltotriose on BAM3. Maltotriose (G3) was added to the assay mix at the concentrations shown and maltose as the reaction product was detected by GC-MS. **A:** BAM3 acting on amylopectin (AP) as substrate, **B:** Barley BAM acting on AP, **C:** BAM3 acting on G6, **D:** Barley BAM acting on G6. Each value is the mean of three independent replicates (+/- SE) and significant differences determined by the Student’s *t*-test are shown (* P > 0.1; ** P > 0.05; *** P > 0.01).

We tested the effect of maltotriose on the activities of BAM3 and barley β-amylase using AP and G6 as substrates. We observed that BAM3 was strongly inhibited by maltotriose when AP was substrate (more than 50% inhibition by 3 mM maltotriose) and weakly inhibited when G6 was substrate (25% inhibition by 10 mM maltotriose) (Fig 5A and 5C). In contrast, barley β-amylase showed little inhibition by maltotriose with AP or G6 as substrates (Fig 5B and 5D). We consider the maltotriose inhibition of BAM3 acting on AP to be highly relevant because this enzyme binds to starch and probably acts primarily on AP rather than small soluble glucans during normal starch breakdown at night [16]. In contrast barley β-amylase is a secreted extracellular enzyme that probably acts on glucans after they have been solubilised by endoamylases [33]. Therefore we consider the inhibition of BAM3 by maltotriose to be of potential biological importance.

**Fig 6:**
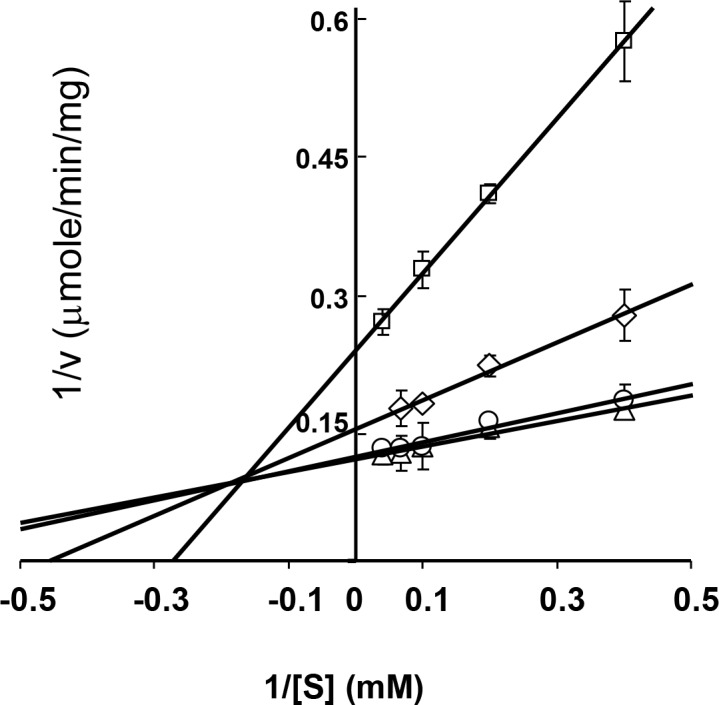
The kinetic parameters of maltotriose inhibition of BAM3. BAM3 kinetics were measured in the presence of maltotriose as an inhibitor using G6 as a substrate. Lineweaver-Burk and kinetic data were generated by the Sigma-enzyme kinetic program.

We investigated the kinetic parameters of maltotriose inhibition of BAM3 using G6 as substrate rather than AP, because AP concentration is difficult to define in molar terms, and difficult to control because it has to be dissolved in hot buffer then diluted and cooled for use which can result in some re-precipitation, depending on concentration and amounts of other assay constituents. We assayed BAM3 at G6 concentrations from 2.5 mM to 25 mM in the presence of maltotriose at concentrations of 3 mM, 10 mM and 30 mM, then carried out a Lineweaver-Burk analysis (Fig 6). The data do not fit to either competitive or non-competitive inhibition by maltotriose, but fit well to mixed-inhibition (R^2^ = 0.97). The K_i_ for maltotriose was 7.5 mM. We would expect the K_i_ to be lower with AP is substrate (Fig 5). The mixed-inhibitor characteristics imply that maltotriose is not competing with substrate for the active site but is binding elsewhere on BAM3. Since BAM3 is known to be a starch-binding protein [16], it might be feasible that maltotriose could bind at the starch-binding site and so inhibit BAM action. We should emphasise that such interpretations are tentative, since kinetic analysis of this enzyme is complex. It can potentially act on a range of different substrates (linear, branched, soluble and insoluble, with varied degree of polymerisation) and produce at least two products (maltose and maltotriose) in different stoichiometric amounts and each with different effects on the enzyme.

## Discussion

We doubt that the small effects of Ca^2+^ and Mg^2+^ on BAM3 activity will play a significant role in the regulation of its activity. The free Mg^2+^ concentration in the stroma of spinach chloroplasts has been estimated to be 0.5 mM in the dark and 2.0 mM in the light [21] which would affect BAM3 activity very little. The free Ca^2+^ concentration has been estimated to be approximately 5 μM in spinach chloroplasts in the dark [22]. Transient increases in stromal Ca^2+^ are observed upon transition from light to dark [34], but it is difficult to see how this would regulate BAM3 activity throughout the night. BAM3 appears to be highly responsive to redox conditions but it is not known if this could provide a fine control mechanism *in vivo*. Many stromal enzymes require to be reduced for maximum activity, including enzymes of starch synthesis (eg AGPase) and breakdown (eg GWD1 and BAM1) so any such control is likely to be quite complex, for example involving specific thioredoxins [7, 27, 35, 36].

Our finding that maltotriose inhibits BAM3 activity raises the possibility that this could provide a mechanism for the flexible control of starch breakdown at night. With AP as substrate, we observed that BAM3 specific activity was inhibited 50% by 3 mM maltotriose and 80% by 10 mM maltotriose. It is difficult to know precisely the concentration of maltotriose in the chloroplast but the K_m_ (maltotriose) of pea chloroplast DPE1 was measured as 3.3 mM [37] implying that it may be in the low mM range. The concentration of maltotriose in Arabidopsis leaves at night has been measured in the range 33 to 70 μg per g fresh weight [18]. If we assume all the maltotriose to be in the chloroplast [18] and if the volume of chloroplast stroma is 10% that of the whole leaf (probably an over-estimate), the concentration of maltotriose in the chloroplast would be in the range 0.6 to 1.4 mM at night. The maltotriose concentration in the *dpe1* mutant calculated in the same way would be between 1.0 mM and 4.6 mM during the night [18]. We therefore believe that endogenous levels of maltotriose could be sufficiently high to regulate BAM3 activity and hence rate of starch breakdown. We consider the fact that BAM3 is inhibited by maltotriose whereas the non-plastid barley β-amylase is relatively insensitive, is consistent with a functional role for maltotriose inhibition in transitory starch breakdown.

Further research will be required to characterise the maltotriose inhibition in detail. It may be valuable to study the properties of non-plastid β-amylases in Arabidopsis for comparison. One of the challenges of investigating the kinetic and regulatory properties of enzymes acting on α-1-4, glucans is that their true substrates are either poorly defined or are not readily amenable to investigation. We believe that it will be particularly valuable to investigate possible regulatory effects of maltotriose on BAM3 in a reconstituted cell-free system employing starch granules together with GWD1, PWD, DSP and ISA3 [12]. If this is carried out in the presence and absence of added maltotriose and DPE1 it could provide a direct test of our hypothesis.

**Fig 7:**
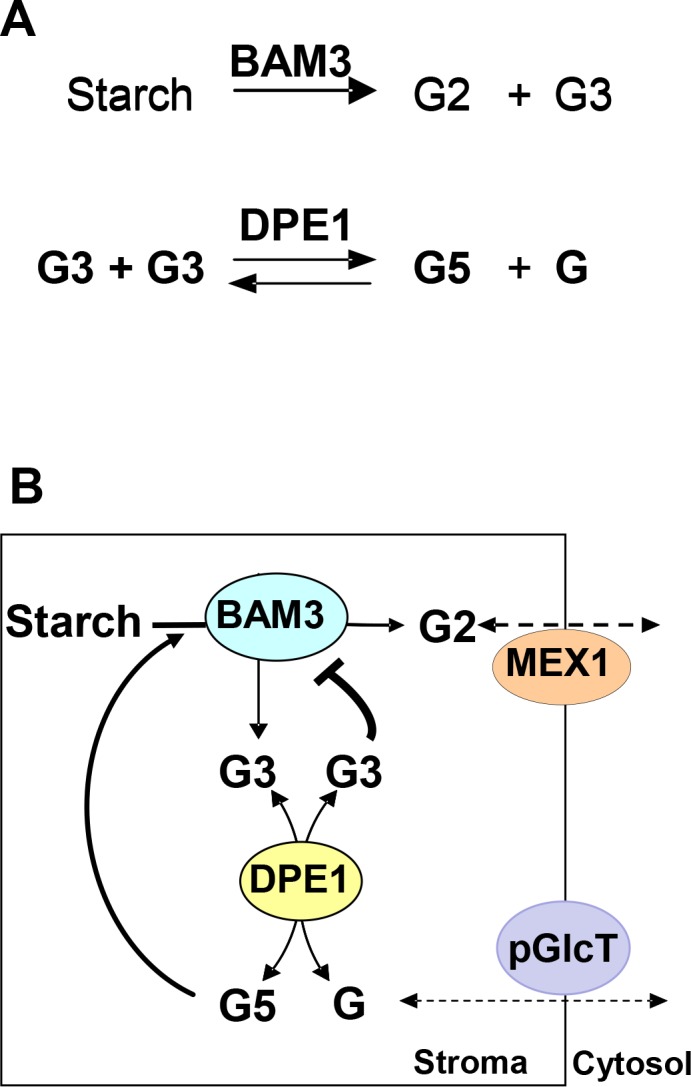
Model for control of starch breakdown by maltotriose and DPE1. **A:** Key enzyme reactions (BAM3 and DPE1) in the model. **B:** Schematic diagram of starch breakdown controlled by maltotriose (G3) and DPE1. The rate at which glucose (G) is exported from the chloroplast determines the stromal concentration of G and hence the G3 concentration. High [G] and hence high [G3] concentration inhibits BAM3 and therefore slows the rate of starch breakdown.

Since maltotriose is one of the products of starch breakdown, it could exert feedback inhibition on BAM3 to regulate the rate of starch breakdown at night. These observations could explain why starch breakdown is inhibited in mutants (eg *dpe1*) that accumulate maltotriose [18]. We hypothesise that the greater the rate at which maltotriose is metabolized for plastid export and cellular metabolism, the lower its concentration in the chloroplast will tend to be, and the faster the rate of starch breakdown can be (Fig 7).

The concentration of maltotriose is determined by DPE activity, which is in turn influenced by the stromal concentration of glucose since it catalyses a fully reversible reaction [18, 38]. Since the plastid glucose transporter pGlcT is a uniporter catalysing facilitated diffusion between stroma and cytosol [39, 40], changes in concentration of glucose in the cytosol are expected to lead to concomitant changes in concentration of plastid glucose and hence maltotriose, leading to compensatory changes in BAM3 activity. In this way, changes in the rates of consumption of sugars in photosynthetic cells and their export to non-photosynthetic tissues could lead to changes in rates of plastid starch mobilisation.

Such a simple model could only provide a part of the control mechanism, because it does not explain how the rate of starch breakdown at night is precisely timed to match the duration of the night [1–5]. It raises an important question as to whether the rate of starch breakdown is ultimately controlled by the chloroplast or by the rate of carbon consumption and export by the cell. In one model it is proposed that the cell conducts an arithmetic division using values for the mass of starch available and the duration of the anticipated night, and then sets an appropriate linear rate for the breakdown of the starch [6]. This model requires a continual and complex ‘recalculation’ of rates of hydrolytic activity relative to substrate availability because the surface area of the substrate (starch granule) declines continuously and the surface-area-to-mass ratio is non-linear. An alternative and perhaps more realistic model assumes that the control is exerted through a readjustment of cellular metabolism by the circadian clock, and that starch breakdown responds to cellular demand. In this case, feedback regulation such as we propose for maltotriose action on BAM3 could provide a suitable mechanism.

Our model implies an important role for pGlcT in the control of starch breakdown, yet a mutant lacking this transporter grows normally and has a relatively normal starch content through the diurnal cycle [41]. However the great majority of carbon is exported from the plastid as maltose, while maltotriose can be continuously converted to maltose by the combined actions of DPE1 and BAM3. Although the concentration of maltotriose in mutants lacking pGlcT has not been reported, we would not expect it to be very high, but rather our model would predict that the mutant might be impaired in adjustment of rates of starch breakdown to changes in photoperiod. Importantly, when pGlcT and MEX1 are both absent, starch breakdown and plant growth are more severely impaired than in a *mex1* single mutant [41]. Likewise a *dpe1 mex1* double mutant is more severely impaired than *mex1* [42], consistent with our hypothesis that DPE1 and pGlcT operate in a common pathway that is not essential but is important for effective control under certain conditions.

Carbon from maltose can be stored in cytosolic heteroglycan potentially acting as a kind of energy buffering system [43, 44]. In contrast some carbon from maltotriose is exported as glucose and might be used directly in metabolism or might have a signalling role such as that mediated by hexokinase [45]. Thus we could imagine an important regulatory role for glucose as both an indicator of energy demand by the cell and perhaps the plant as a whole, and as an indirect regulator of the rate of chloroplast starch breakdown. Reduced demand for glucose could lead to build up of maltotriose and feedback inhibition of starch breakdown.

## Supporting information

S1 Fig Effect of disaccharides and trisaccharides on BAM3 activity. Assays were carried out using the Betamyl method. Each disaccharide and trisaccharide was added at the start of the reaction. **A:** Effects of 5 mM disaccharide and trisaccharide on BAM3. **B:** Effects of isomaltose in the range 5 mM to 50 mM on BAM3. Each value is the mean of three independent replicates (+/- SE). No significant differences were revealed by the Student’s *t*-test.
(TIF)

S2 Fig Effect of glucose on BAM3 and barley BAM activity. Glucose was added to assays at the concentrations shown, and maltose production was measured by GC-MS. **A:** BAM3, **B:** Barley BAM.
(TIF)
