# Supplementary figures and images for: Inhibition of Arabidopsis chloroplast β-amylase BAM3 by maltotriose suggests a mechanism for the control of transitory leaf starch mobilisation
Source: PLoS One. 2017 Feb 22;12(2):e0172504. doi: 10.1371/journal.pone.0172504 (PMC5321445; doi:10.1371/journal.pone.0172504)

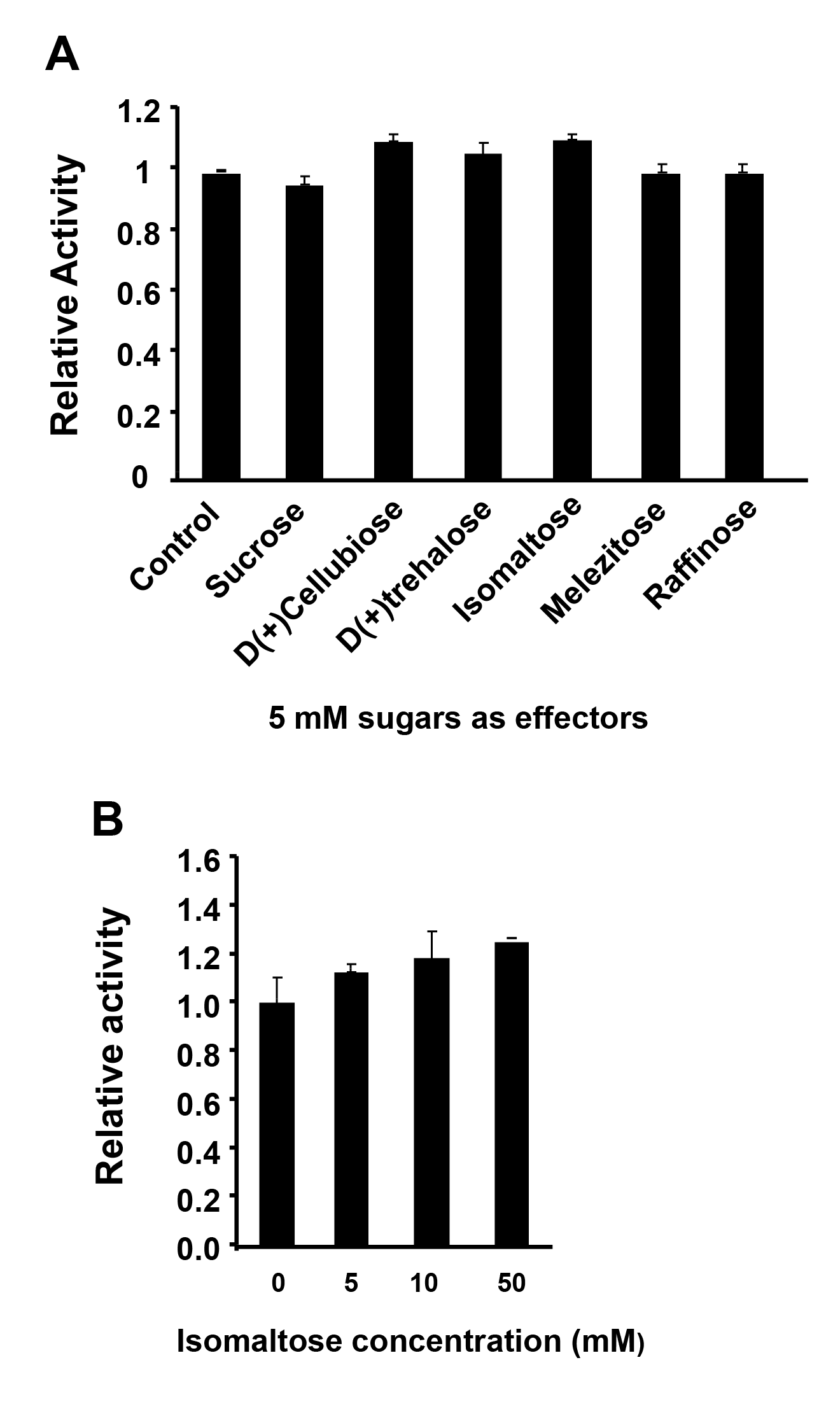

Supplement: S1 Fig — Assays were carried out using the Betamyl method. Each disaccharide and trisaccharide was added at the start of the reaction. A: Effects of 5 mM disaccharide and trisaccharide on BAM3. B: Effects of isomaltose in the range 5 mM to 50 mM on BAM3. Each value is the mean of three independent replicates (+/- SE). No significant differences were revealed by the Student’s t-test. (TIF) [file pone.0172504.s001.tif]

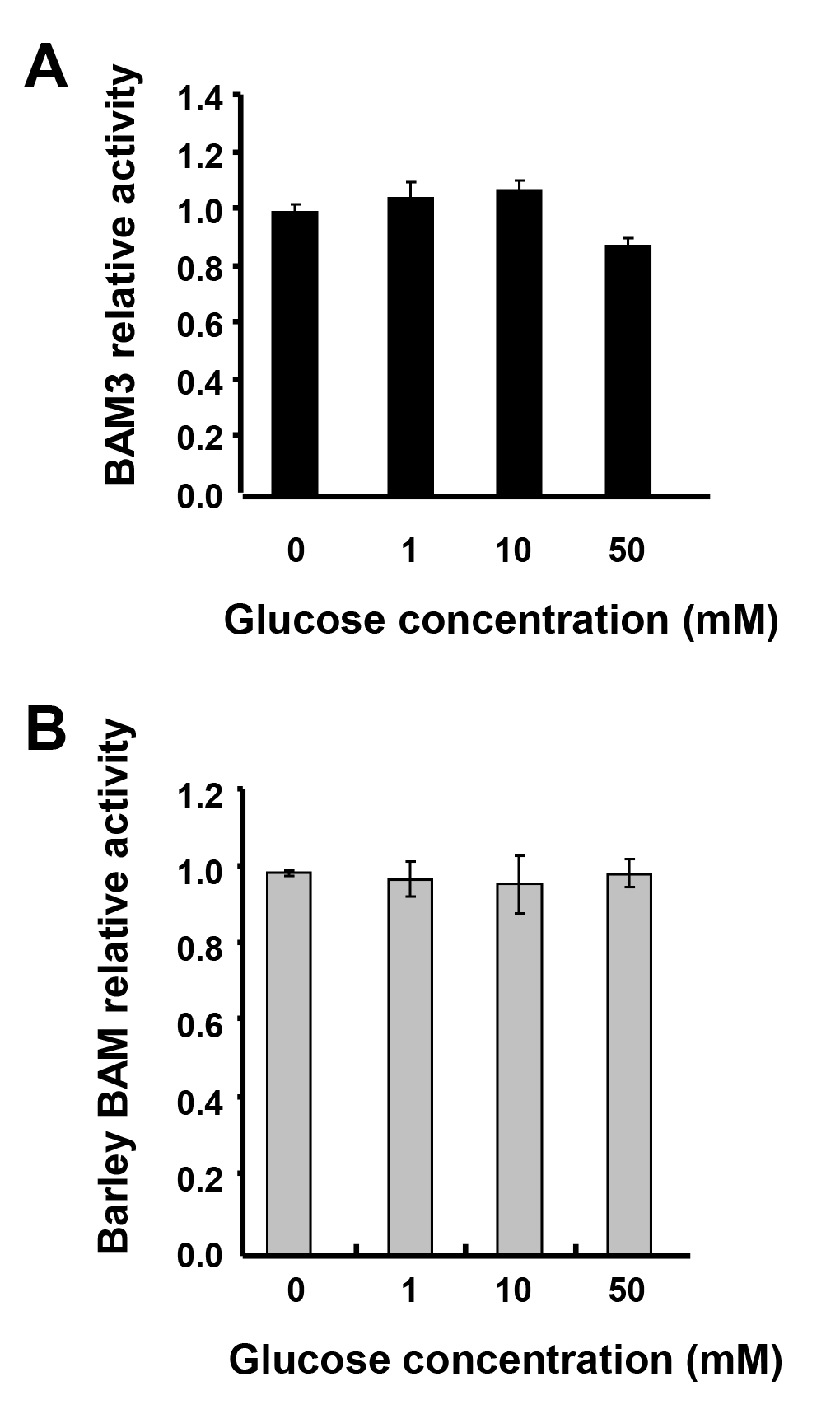

Supplement: S2 Fig — Glucose was added to assays at the concentrations shown, and maltose production was measured by GC-MS. A: BAM3, B: Barley BAM. (TIF) [file pone.0172504.s002.tif]
